# AI System Usability and Learning Engagement in Nursing Simulation: Cross‐Sectional Statistical Indirect Associations Through Extraneous Cognitive Load and Flow Experience

**DOI:** 10.1002/nop2.70855

**Published:** 2026-09-18

**Authors:** Mengjiao Liu, Ping Zhang, Yeqing Wu, Lu Pan, Lan Li

**Affiliations:** ^1^ Trauma Center The Second Xiangya Hospital, Central South University Changsha Hunan China; ^2^ Clinical Nursing Teaching and Research Section The Second Xiangya Hospital, Central South University Changsha Hunan China; ^3^ Department of Neurosurgery The Second Xiangya Hospital, Central South University Changsha Hunan China; ^4^ Department of Cardiovascular Medicine The Second Xiangya Hospital, Central South University Changsha Hunan China; ^5^ Department of Pathology The Second Xiangya Hospital, Central South University Changsha Hunan China

**Keywords:** artificial intelligence, cognitive load, flow experience, learning engagement, nursing education, structural equation model, system usability

## Abstract

**Introduction:**

The association between artificial intelligence simulation‐system usability and nursing students' learning engagement remains unclear.

**Aim(s):**

To examine cross‐sectional indirect associations linking system usability, extraneous cognitive load, flow, and learning engagement, and explore moderation by digital readiness among nursing students.

**Methodology:**

The hospital research team conducted an online cross‐sectional survey of 2016 nursing students. Teachers at 16 colleges provided only voluntary, public‐interest assistance by forwarding the survey link; neither the colleges nor their personnel were participating research institutions. Established measures, structural equation modelling, bootstrap tests, and multi‐group analysis were used.

**Results:**

Higher usability was associated with lower extraneous load and higher engagement; lower load with greater flow, and greater flow with engagement. The sequential indirect association was supported. The load‐flow association was stronger at lower digital readiness. Concurrent measurement precludes causal inference.

**Conclusion:**

Usability, extraneous cognitive load, flow, and engagement showed theoretically ordered cross‐sectional associations. Longitudinal or experimental studies are needed to test temporal ordering.

**Review Methods:**

Not applicable to this empirical cross‐sectional study.

**Data Sources:**

From August to December 2025, the hospital research team conducted an online anonymous survey of 2016 nursing students. Teachers at 16 colleges only assisted in forwarding the questionnaire link and were not participating research institutions.

**Implications for the Profession and/or Patient Care:**

Accessible interfaces and differentiated support should be considered, especially for less digitally ready students.

**Impact:**

The study addressed the gap in understanding the psychological pathways (cognitive load and flow) that link the usability of AI systems to nursing students' engagement in virtual simulation. Higher usability was associated with lower load, greater flow, and higher engagement; the load‐flow association varied by digital readiness. The findings may inform accessible simulation‐system design for diverse nursing students.

**Reporting Method:**

The Strengthening the Reporting of Observational Studies in Epidemiology checklist was followed.

**No Patient or Public Contribution:**

No patient or members of the public were involved because participants were nursing students.

## 1 Introduction

Artificial intelligence (AI)‐driven virtual simulation systems have become an important component of contemporary nursing education, providing a safe environment for bridging theoretical knowledge and clinical practice (Narayanan et al. 2023; Bai et al. 2022). Globally, research on AI in nursing practice and education has expanded rapidly over the past decade, with annual publications increasing from 38 in 2014 to 563 in 2024 (Badawy et al. 2025). In parallel, virtual simulation has attracted increasing attention in nursing education, and a national web‐based survey in China showed that nursing interns had generally positive attitudes toward diabetes virtual simulation teaching applications, although actual understanding and use remained limited (Liu et al. 2021). However, the adoption of educational technology alone does not guarantee effective learning outcomes. Previous studies have shown substantial variability in students' digital learning experiences, highlighting system usability as a critical antecedent of technology acceptance and learning‐related outcomes (Chuenyindee et al. 2022; Haanes et al. 2024; Alqurni 2023). For instance, Haanes et al. (2024) reported that nursing students' experiences with e‐learning varied considerably, with benefits such as flexibility coexisting alongside challenges related to technical difficulties and reduced interaction. Similarly, Alqurni (2023) found that key usability factors of e‐learning software significantly affected learner satisfaction among university students, further underscoring the importance of system design quality. Despite this recognition, the cross‐sectional cognitive and experiential associations linking system usability with learning engagement remain insufficiently understood.

Cognitive load theory offers a useful framework for explaining this process. Given the limited capacity of working memory (Hanham et al. 2023). Poorly designed AI systems may impose excessive extraneous cognitive load, diverting learners' cognitive resources toward managing system interaction rather than processing core nursing knowledge (Storck et al. 2025; Zhang et al. 2023). Such system‐induced load may also impede the emergence of flow experience. Flow theory suggests that optimal engagement occurs when learners can concentrate fully under conditions of balanced challenge and minimal distraction (Gold and Ciorciari 2020). Accordingly, greater usability may be associated with lower extraneous cognitive load and greater flow, which may in turn be associated with learning engagement.

However, few studies have simultaneously integrated extraneous cognitive load and flow experience within a single framework to examine their sequential mediating roles between system usability and learning engagement. In addition, empirical evidence from large samples remains limited regarding whether digital readiness moderates students' adaptation to AI‐based learning systems.

Therefore, this study used a large‐sample cross‐sectional survey to examine a hypothesized sequential statistical indirect‐association model. We hypothesized that greater AI system usability would be associated with lower extraneous cognitive load, greater flow experience, and higher learning engagement, and that digital readiness would moderate the load‐flow association. These hypotheses concern contemporaneous associations and do not establish temporal ordering or causation.

## 2 Materials and Methods

### 2.1 Study Participants

**FIGURE 1:**
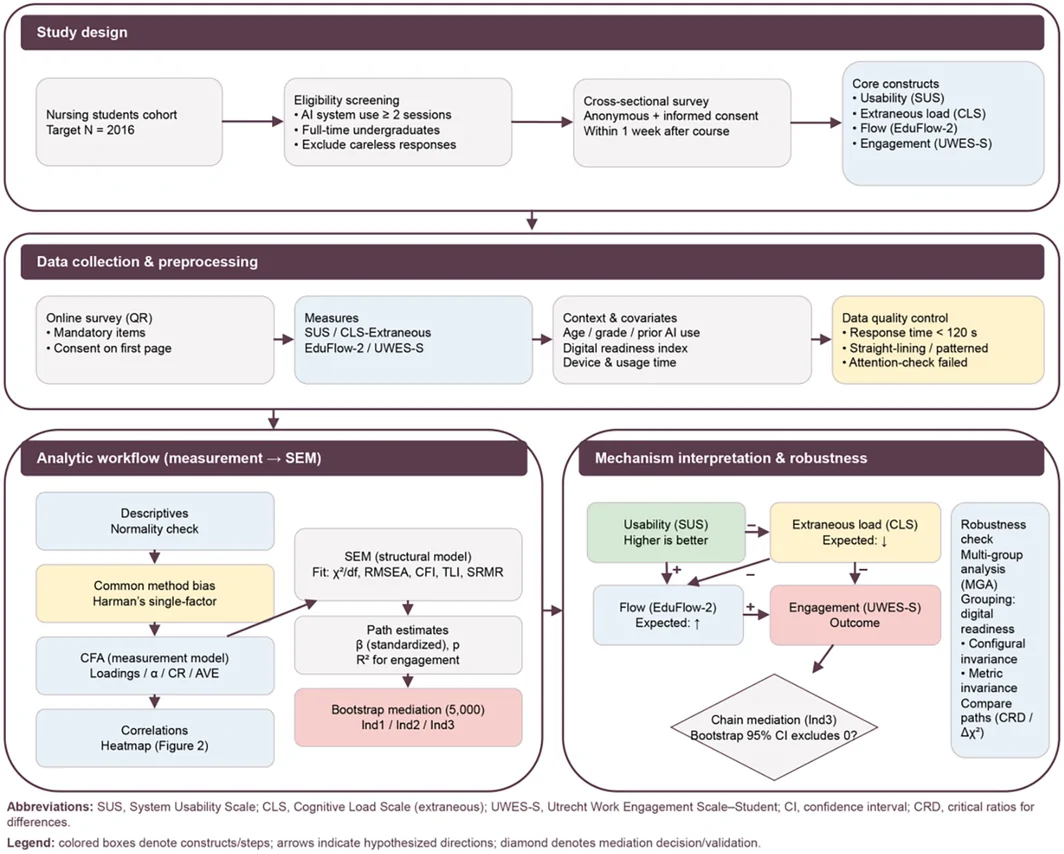
The methodological workflow and hypothesized chain mediation model. The diagram illustrates the study design, analytical strategy, and hypothesized cross‐sectional statistical associations. Arrows indicate the direction of model specification and do not establish temporal ordering or causation. Legend: Colored boxes denote constructs/steps; arrows initiate hypothesized directions; diamond denotes mediation decision/validation. Abbreviations: CI, confidence interval; CLS, Cognitive Load Scale (extraneous); CRD, critical ratios for differences; SUS, System Usability Scale; UWES‐S, Utrecht Work Engagement Scale‐Student.

This hospital‐led study employed a cross‐sectional online anonymous survey design. The overall design framework, data collection process, and analytical strategy are detailed in Figure 1. From August to December 2025, teachers at 16 medical colleges across central, eastern, and western China, including 8 undergraduate universities and 8 vocational medical colleges, provided voluntary, public‐interest assistance by forwarding the standardized survey link or QR code to nursing students. These colleges were not participating research institutions: their teachers did not identify, screen, enrol, or follow individual participants and had no role in informed‐consent administration, data collection, sample management, data access, or data analysis. This purposive dissemination approach does not establish national representativeness. Eligible participants were full‐time associate degree, undergraduate, or professional master's degree nursing students who self‐reported having used the target adaptive virtual simulation system (e.g., virtual simulation experiment platform or intelligent nursing training system) at least twice in current‐semester courses, provided online informed consent, and participated voluntarily. Exclusion criteria were: (1) students who reported not participating in courses involving the system because of leave, internship, or other reasons; (2) questionnaires completed in less than 120 s; (3) questionnaires showing obvious patterned responses (e.g., all items selecting the same option or a wave pattern); and (4) an incorrect response to the embedded attention‐check item.

#### 2.1.1 Sample Size Determination

The sample size was determined a priori based on recommendations for structural equation modelling (SEM). Kline (2016) recommended a minimum sample size of 200 for stable parameter estimates in SEM, while other scholars have suggested a subject‐to‐parameter ratio of at least 10:1 to 20:1 (Wolf et al. 2013). Given that the hypothesized model contained 31 observed indicators and multiple latent constructs, a minimum of 310–620 participants was required. To improve precision and ensure adequate power for the planned multi‐group comparisons, the hospital research team sought a substantially larger sample. The final sample of 2016 valid anonymous responses exceeded these minimum thresholds and provided robust statistical power for the planned individual‐level analyses.

#### 2.1.2 Sample Composition and Representativeness

The sample comprised 1580 undergraduate students (78.37%), 380 associate degree students (18.85%), and 56 postgraduate students (2.78%). This distribution describes the participating sample and should not be interpreted as the national enrolment structure of nursing programmes in China (Amin et al. 2025). The inclusion of all three educational levels was intentional: the postgraduate cohort, though small, was included because their training programs mandatorily incorporated advanced clinical simulation modules on the same AI platform, making their learning experiences directly relevant to the research questions. To account for potential differences associated with the unequal distribution of educational levels, age, year of study, and education level were included as covariates in the structural equation model.

Regarding the 9 first‐year students (0.45% of the sample), eligibility was determined from their responses to the prespecified anonymous screening item confirming use of the target adaptive virtual simulation system at least twice during the current semester. These students were enrolled in programmes in which the platform was integrated into first‐year courses (e.g., Fundamentals of Nursing). No course‐enrolment lists, platform‐use logs, student names, student numbers, platform account identifiers, or other individual records were requested from or provided by the colleges. The anonymous questionnaire dataset could not be linked to school records or platform‐use data.

### 2.2 Ethical Considerations

This hospital‐led study was conducted in accordance with the Declaration of Helsinki and was approved by the Ethics Committee of the Second Xiangya Hospital, Central South University (Approval No. LYEC2025‐0101; approval date: 2025.4.23). This committee served as the sole ethics review body for the study. Before survey dissemination, the hospital research team contacted the designated faculty member or relevant academic unit at each of the 16 colleges and obtained permission to circulate the standardized study invitation and survey link through the relevant student communication channels. The colleges' involvement was limited to this dissemination role. Beyond forwarding the study invitation, college personnel did not implement the study protocol, determine eligibility, recruit or enroll participants, obtain informed consent, administer the questionnaire, access questionnaire responses, manage data, or conduct data analyses.

All participants read an online information sheet and provided electronic informed consent before completing the questionnaire. The questionnaire was anonymous and collected no names, student numbers, platform account identifiers, college or school identifiers, or other directly identifying information. Individual platform‐use logs were neither obtained nor linked to questionnaire responses. Faculty members who forwarded the survey link could not view individual participation, consent, response status, or completed questionnaires. Only the hospital research team had access to, managed, and analysed the anonymous dataset.

Students were informed that participation was voluntary, that they could discontinue the questionnaire at any time before submission, and that participation or non‐participation would have no effect on course grades, clinical assessments, academic standing, or access to educational resources. The data were used solely for this study and stored securely by the hospital research team.

A total of 2030 questionnaires were returned. After excluding invalid questionnaires based on preset data quality standards, 2016 valid samples were included.

### 2.3 Educational Intervention and AI System

The teaching system used in this study was a nursing virtual simulation platform developed by Beijing Oubeier Software Technology Development Co. Ltd., including the Comprehensive Training System for Basic Nursing Skills and the Critical Care Virtual Simulation System. The platform supports multi‐terminal access through individual student accounts and was used primarily for post‐class independent practice in Fundamentals of Nursing and Medical‐Surgical Nursing. Students completed scenario‐based tasks involving nursing assessment, clinical decision‐making, and intervention implementation.

The platform (Version 3.2, launched in 2024) included more than 50 nursing‐skill modules, with each simulation task typically requiring 20–30 min. During simulation, the system recorded students' operational steps, selected decision pathways, and response times. These inputs were compared with predefined task rules and performance thresholds to trigger branch‐specific feedback, recommend repeated practice, and adjust subsequent scenario difficulty within the preset module structure. These records were generated by the platform during routine teaching; however, no individual‐level platform logs were obtained, extracted, analysed, or linked to questionnaire responses for this study. Eligibility was determined solely from the prespecified anonymous self‐report screening item.

Accordingly, the adaptive function evaluated in this study was rule‐based: it used prespecified procedural rules, decision branches, and performance thresholds rather than a generative artificial intelligence model or autonomous model updating. The system provided individualized learning profiles based on recorded performance and operation history, allowing students to identify areas for further practice. Therefore, the platform is described in this study as an adaptive virtual simulation system rather than an artificial intelligence or machine‐learning system.

The platform operated in both practice and assessment modes. The assessment mode combined procedural‐accuracy checks with competency‐oriented scoring and generated individual performance reports for learning monitoring. These system‐generated reports were not used as outcome variables in the present survey.

### 2.4 Data Collection

Data collection was conducted within 1 week after completion of the relevant nursing simulation courses. The hospital research team administered a standardized online questionnaire through the Wenjuanxing platform. Teachers at 16 colleges only provided voluntary, public‐interest assistance by forwarding the uniform questionnaire link or QR code in class group chats; the colleges were not research sites or participating institutions. The teachers did not identify, screen, recruit, enroll, monitor, or follow individual participants; they did not administer consent, collect or manage samples or data, access the dataset, or conduct analyses. The first page of the questionnaire contained the online informed‐consent form, which explained the research purpose, voluntary participation, anonymity, and research‐only use of data. No names, student numbers, platform account identifiers, college or school identifiers, or other directly identifying information were collected. To minimize item‐level missing data, the online questionnaire required completion of all items. Data quality was controlled using a completion‐time threshold, patterned‐response detection, and an attention‐check item. The attention‐check item read: “To prove you are reading carefully, please select ‘Strongly Disagree’ for this item.” Responses failing this item were automatically marked as invalid. Because academic calendars varied between August and December 2025, the link was forwarded during a 1‐week survey window after course completion. Teachers who forwarded the link could not view individual participation or response status, and participation had no effect on grades or course evaluation.

#### 2.4.1 Briefing for Questionnaire‐Link Disseminators

Before the survey link was forwarded, the hospital research team supplied teachers with a brief standardized written message and the uniform link or QR code. The message explained that forwarding was voluntary, limited to dissemination assistance, and did not make the teacher, college, or school a participating research institution. Teachers were instructed not to answer research questions on behalf of the hospital research team, identify or screen students, obtain consent, monitor participation or completion, access response data, manage samples, or undertake data analysis. Students with questions were directed to the hospital research team. This briefing was solely intended to ensure neutral and consistent link forwarding and to protect the anonymity and voluntariness of participation.

### 2.5 Measurement Tools

All measurement tools used in this study are internationally recognized, standardized instruments with well‐established psychometric properties. The details of each tool, including scoring interpretation, item directionality, and reliability evidence, are described below.

#### 2.5.1 Demographics and Digital Readiness

Information collected included participants' age, gender, year of study, and hometown (urban/rural). Additionally, participants' digital readiness was assessed. This index was synthesized from two parts: (1) Prior AI experience, defined as the frequency of using AI‐based learning tools before the current semester (no prior use = 0 points, used 1–2 times = 1 point, used ≥ 3 times = 2 points), consistent with the categories reported in Table 1; (2) Self‐perceived digital competence (1 = very poor, 5 = very good). The scores from both parts were summed (total score range 1–7), with higher scores indicating greater readiness for digital learning. Notably, prior AI experience captures students' exposure to AI‐based learning tools before the current semester and is therefore distinct from the inclusion criterion, which required at least two uses of the target AI teaching system during the current semester; accordingly, the students categorized as having no prior experience were first‐time adopters in the current semester who nevertheless satisfied the inclusion criterion. This was a study‐specific exploratory index rather than a validated psychometric scale. To avoid conceptual overlap, prior AI experience was used only as a component of the digital‐readiness index and was not entered as a separate covariate.

**TABLE 1:** Demographic characteristics of the participants (*n* = 2016).

Variable	Category	*n*	%
Gender	Male	333	16.52
	Female	1683	83.48
Education level	College	380	18.85
	Undergraduate	1580	78.37
	Postgraduate	56	2.78
Age group (years)	≤ 18 years	216	10.71
	19–24 years	1782	88.39
	≥ 25 years	18	0.89
Year of study	Year 1	9	0.45
	Year 2	1512	75
	Year 3	423	20.98
	Year 4	72	3.57
Hometown	Urban	540	26.79
	Rural	1476	73.21
Prior AI experience (before current semester)	No experience	207	10.27
	1–2 times	666	33.04
	≥ 3 times	1143	56.7
Perceived digital competence	Very poor	36	1.79
	Poor	135	6.7
	Average	936	46.43
	Good	657	32.59
	Very good	252	12.5

*Note:* The distributions of the continuous core variables meet the normality requirements for SEM analysis (|Skewness| < 3, |Kurtosis| < 10).

#### 2.5.2 System Usability Scale (SUS)

The SUS (Zhang et al. 2023) was used to assess students' perceived usability of the AI system. The SUS is a widely used, standardized, and technology‐independent questionnaire originally developed by Brooke (1996). The scale contains 10 items (e.g., “I think that I would like to use this system frequently”), rated on a 5‐point Likert scale (1 = strongly disagree, 5 = strongly agree). Items 1, 3, 5, 7, and 9 are positively worded, while items 2, 4, 6, 8, and 10 are negatively worded and require reverse scoring. For positively worded items, the score contribution is the scale position minus 1; for negatively worded items, the contribution is 5 minus the scale position. The sum of item scores is then multiplied by 2.5 to produce a total score ranging from 0 to 100, where higher scores indicate better perceived usability. A SUS score above 68 is generally considered above average usability (Bangor et al. 2008). The tool has demonstrated excellent reliability and validity in previous educational technology studies, with Cronbach's *α* values consistently reported above 0.80 across diverse contexts (Bangor et al. 2008; Khan et al. 2025). In the current study, the Cronbach's *α* was 0.899 and the composite reliability (CR) was 0.917. For SEM, the value of 3.256 reported in Table 2 represents the reverse‐scored 1–5 item mean; using the conventional SUS transformation, this corresponds to 56.4 on the 0–100 scale.

**TABLE 2:** Descriptive statistics and correlations among core variables (*n* = 2016).

Variable	Mean	SD	1	2	3	4
1. Usability	3.256	0.646	1	−0.409	0.218	0.284
2. Extraneous load	3.004	0.684	−0.409	1	−0.394	−0.257
3. Flow	3.373	0.597	0.218	−0.394	1	0.468
4. Engagement	3.485	0.58	0.284	−0.257	0.468	1

Abbreviation: SD, standard deviation.

#### 2.5.3 Extraneous Cognitive Load Scale

The Cognitive Load Scale (CLS) developed by Leppink et al. (2013) was used. The CLS is a standardized, validated instrument designed to measure three types of cognitive load (intrinsic, extraneous, and germane) in learning contexts (Leppink et al. 2013). This study focused on the extraneous cognitive load dimension, consisting of 3 items (e.g., “The instructions and explanations in the system were very unclear”). All three items in the extraneous cognitive load subscale are negatively framed (i.e., higher scores reflect greater ineffective cognitive burden imposed by the instructional design). Items were rated on a 10‐point Likert scale (0 = not at all the case, 10 = completely the case), with higher scores indicating greater ineffective cognitive load imposed by system design. The original validation study reported satisfactory internal consistency for the extraneous load subscale (Cronbach's *α* = 0.77–0.82 across different samples) (Leppink et al. 2013). A subsequent systematic meta‐analysis by Krieglstein et al. (2022) confirmed the reliability and validity of the CLS across diverse educational settings. In the current study, the Cronbach's *α* was 0.749 and the CR was 0.857.

#### 2.5.4 Flow Experience Scale

Flow experience was assessed using an adapted nine‐item version of the Flow in Education Scale (EduFlow‐2) (Heutte et al. 2021). The original EduFlow‐2 is a 12‐item multidimensional instrument developed and validated by Heutte et al. (2021). To assess flow experience in educational settings. It comprises four three‐item dimensions: cognitive control, immersion and time transformation, loss of self‐consciousness, and autotelic experience. In the present study, nine items were selected because they were considered most relevant to students' task‐based learning experiences in nursing virtual simulation. The three non‐retained items were omitted to reduce respondent burden and because they were considered less directly applicable to the specific simulation‐learning context. Accordingly, the measure used in this study should be interpreted as an adapted nine‐item EduFlow‐2 measure rather than the complete original instrument.

The retained items were rated on a 7‐point Likert scale ranging from 1 (strongly disagree) to 7 (strongly agree), with higher scores indicating a stronger flow experience during nursing simulation learning. All retained items were positively worded and no reverse scoring was required. The Chinese wording of the selected items was reviewed for clarity and contextual appropriateness for nursing education before administration. The content relevance of the adapted items was assessed in relation to the learning objectives and task characteristics of the simulation courses. Use of the scale followed the applicable terms of the original instrument.

In the present sample, the adapted nine‐item scale demonstrated acceptable internal consistency (Cronbach's *α* = 0.889), composite reliability (CR = 0.910), and convergent validity (average variance extracted [AVE] = 0.530). Standardized factor loadings ranged from 0.719 to 0.742. These results provide preliminary support for the reliability and convergent validity of the adapted measure in this study; however, they do not replace validation of the complete original EduFlow‐2 or formal validation of a shortened version in other nursing education samples.

#### 2.5.5 Learning Engagement Scale

The 9‐item Utrecht Work Engagement Scale for Students (UWES‐S) (Carmona‐Halty et al. 2019) was used. The UWES‐S is a standardized and internationally validated instrument for measuring academic engagement, originally adapted from the Utrecht Work Engagement Scale by Schaufeli et al. (2006) and further validated by Carmona‐Halty et al. (2019). It includes three dimensions: vigour, dedication, and absorption. Items were rated on a 7‐point Likert scale, with higher scores indicating higher levels of learning engagement. All items are positively worded; no reverse scoring is required. The original validation study reported Cronbach's *α* values of 0.82–0.90 across different cultural samples (Carmona‐Halty et al. 2019; Schaufeli et al. 2006). In the current study, the Cronbach's *α* was 0.876 and the CR was 0.901.

### 2.6 Statistical Analysis

Data were analysed by the hospital research team using IBM SPSS Statistics version 26.0 and R version 4.3.1. CFA and SEM were performed using the lavaan package (version 0.6–17). Scale items were treated as continuous indicators, and robust maximum likelihood estimation (MLR) was used. Continuous variables were summarized as mean ± standard deviation or median (interquartile range), as appropriate, and categorical variables as frequency and percentage. Group comparisons were performed using independent‐samples *t*‐tests or Mann–Whitney *U* tests for continuous variables and *χ* ^2^ or Fisher's exact tests for categorical variables. Common method bias (CMB) was assessed using two complementary approaches: Harman's single‐factor test and the unmeasured latent method construct (ULMC) approach, in which a common method factor was added to the confirmatory factor analysis model and changes in model‐fit indices and substantive factor loadings were examined. Confirmatory factor analysis was conducted to evaluate the measurement model, with reliability and validity assessed using factor loadings, Cronbach's *α*, composite reliability, and average variance extracted. Structural equation modelling was then applied to test the hypothesized relationships. Model fit was evaluated using standard indices (*χ* ^2^/df, RMSEA, CFI, TLI, and SRMR). Mediation effects were examined using a bias‐corrected bootstrap method with 5000 resamples; mediation was considered significant when the 95% confidence interval did not include zero. Age, year of study, and education level were included as covariates; prior AI experience was not entered separately because it formed part of the digital‐readiness index. To examine moderation by digital readiness, participants were initially divided into high‐ and low‐readiness groups using a median split for multi‐group analysis. Although median splits can result in information loss, a supplementary moderation analysis was therefore conducted, in which the standardized continuous digital‐readiness score, extraneous cognitive load, and their interaction term were entered to predict flow experience, followed by simple‐slope analysis at ±1 standard deviation (SD) of digital readiness. Measurement invariance was preliminarily assessed before multi‐group analysis; the values currently reported in Table 7A remain pending verification against the original output and should not be interpreted as fully verified results. Statistical significance was set at *p* < 0.05. Because all questionnaire items were mandatory, retained cases had no item‐level missing data. Because the anonymous questionnaire did not collect college or school identifiers, analyses were conducted at the individual‐respondent level. School personnel had no access to the anonymous dataset and did not participate in data management or analysis.

## 3 Results

### 3.1 Sample Characteristics

A total of 2016 valid questionnaires were analysed. The majority of participants were female (83.48%) and aged 19–24 years (88.39%). The sample comprised undergraduate students (78.37%), college associate degree students (18.85%), and a small cohort of postgraduate students (2.78%). Most students were in their second or third year of study. Regarding prior AI experience, 56.70% had used AI‐based learning systems three or more times. Detailed socio‐demographic data are presented in Table 1.

### 3.2 Descriptive Statistics of the Measurement Tools

As shown in Table 2, the reverse‐scored mean usability item score was 3.256 (SD = 0.646), corresponding to 56.4 on the conventional 0–100 SUS scale. The mean extraneous cognitive load, flow experience, and learning engagement scores were 3.004, 3.373, and 3.485, respectively. The distributions met the stated normality thresholds for subsequent analysis.

### 3.3 Inferential Analysis: Model Testing and Pathways

To understand the relationships between these variables, we first checked the measurement quality. The tools demonstrated excellent reliability and validity (Tables 3 and 4). Common method bias was formally examined using two approaches. Harman's single‐factor test showed that the first unrotated factor explained 28.7% of the total variance, below the 40% threshold. In the ULMC test, adding a common method factor to the measurement model did not substantially improve model fit (ΔCFI = 0.004, ΔRMSEA = 0.002); the method factor accounted for only 4.8% of the total variance, and all substantive factor loadings remained statistically significant after its inclusion, indicating that CMB was not a serious concern in this study. We then conducted correlation and structural equation modelling (SEM) analyses to test our hypotheses in a straightforward manner.

**TABLE 3:** Reliability and convergent validity of the measurement model.

Construct (scale)	Items	Cronbach's *α*	CR	AVE	Factor loading range
Usability (SUS)	10	0.899	0.917	0.526	0.703–0.756
Extraneous load (CLS)	3	0.749	0.857	0.666	0.803–0.828
Flow (EduFlow‐2)	9	0.889	0.91	0.53	0.719–0.742
Engagement (UWES‐S)	9	0.876	0.901	0.502	0.688–0.723

Abbreviations: AVE, average variance extracted; CLS, Cognitive Load Scale; CR, composite reliability; EduFlow‐2, Flow in Education scale; SUS, System Usability Scale; UWES‐S, 9‐item Utrecht Work Engagement Scale for Students.

**TABLE 4:** Discriminant validity (Fornell–Larcker criterion).

	Usability	Extraneous load	Flow	Engagement
Usability	0.725	−0.409	0.218	0.284
Extraneous load	−0.409	0.816	−0.394	−0.257
Flow	0.218	−0.394	0.728	0.468
Engagement	0.284	−0.257	0.468	0.709

*Note:* Diagonal elements are the square roots of the average variance extracted (AVE). These values are greater than the corresponding inter‐construct correlations (off‐diagonal elements), indicating adequate discriminant validity.

**FIGURE 2:**
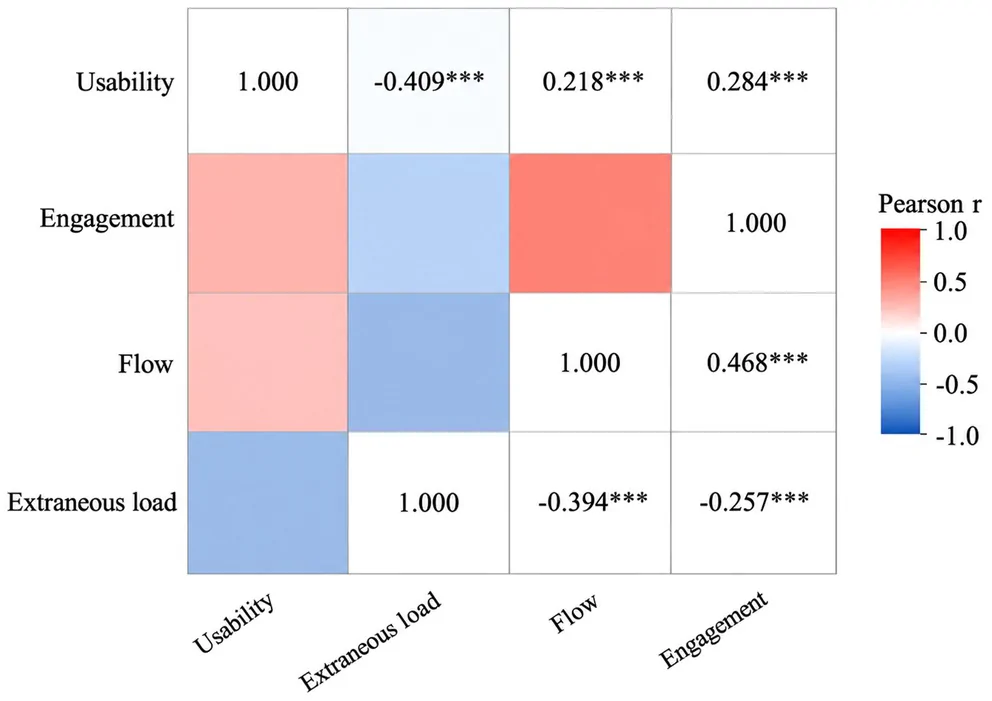
Pearson correlation heatmap among the four core constructs. Colour intensity corresponds to the magnitude of Pearson correlation coefficient r. Red indicates positive correlation and blue indicates negative correlation. ***p < 0.001. *n* = 2016.

Correlation analysis (Table 2 and Figure 2) revealed that a more usable AI system was associated with lower extraneous cognitive load and higher learning engagement. Meanwhile, higher cognitive load was linked to lower flow experience and lower engagement.

**FIGURE 3:**
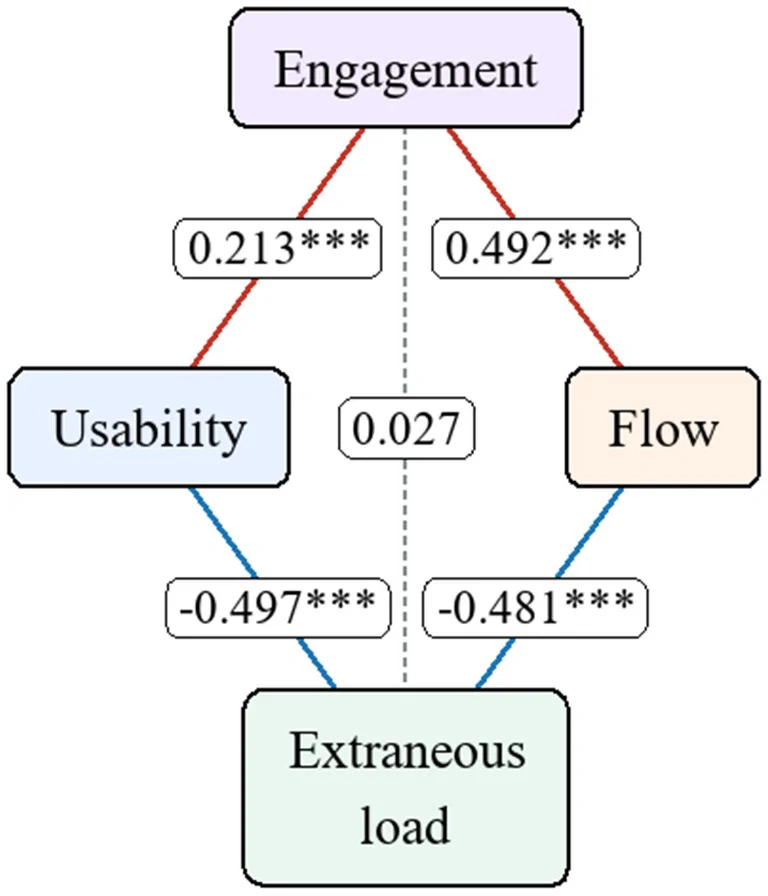
Cross‐sectional structural association diagram. Values in boxes are standardized path coefficients (β). Solid lines represent statistically significant paths; dashed lines represent non‑significant paths. ***p < 0.001. Given the cross‑sectional design, paths reflect statistical associations rather than causal effects. *n* = 2016.

The SEM analysis (Figure 3 and Table 5) showed that greater AI system usability was associated with lower extraneous cognitive load (*β* = −0.497). Greater extraneous cognitive load was associated with lower flow experience (*β* = −0.481), and greater flow was associated with higher learning engagement (*β* = 0.492). System usability also showed a direct positive association with engagement (*β* = 0.213).

**TABLE 5:** Structural model results (standardized association coefficients).

Hypothesis/path	*β* (std. all)	*p*
H1: usability → engagement	0.213	< 0.001
H2: usability → extraneous load	−0.497	< 0.001
H3: extraneous load → flow	−0.481	< 0.001
H4: flow → engagement	0.492	< 0.001
Usability → flow	0.003	0.895
Extraneous load → engagement	0.027	0.417

Bootstrap analysis (Table 6) supported a sequential statistical indirect association from usability through extraneous cognitive load and flow to engagement (indirect association = 0.116). Because all variables were measured concurrently, this result does not establish temporal ordering or causation.

**TABLE 6:** Bootstrap analysis of statistical indirect associations.

Pathway	Std. estimate (*β*)	SE	95% CI lower	95% CI upper	Sig.
Indirect association 1: usability → extraneous load → engagement	−0.013	0.017	−0.045	0.020	NS
Indirect association 2: usability → flow → engagement	0.003	0.013	−0.022	0.030	NS
Indirect association 3: usability → extraneous load → flow → engagement	0.116	0.011	0.095	0.140	***
Direct: usability → engagement	0.212	0.027	0.127	0.266	***
Total effect	0.318	0.023	0.273	0.363	***

*Note:* β = Standardized path coefficient; CI = Confidence interval based on bias‐corrected bootstrap method; ***p < 0.001.

Abbreviation: NS, not significant.

**FIGURE 4:**
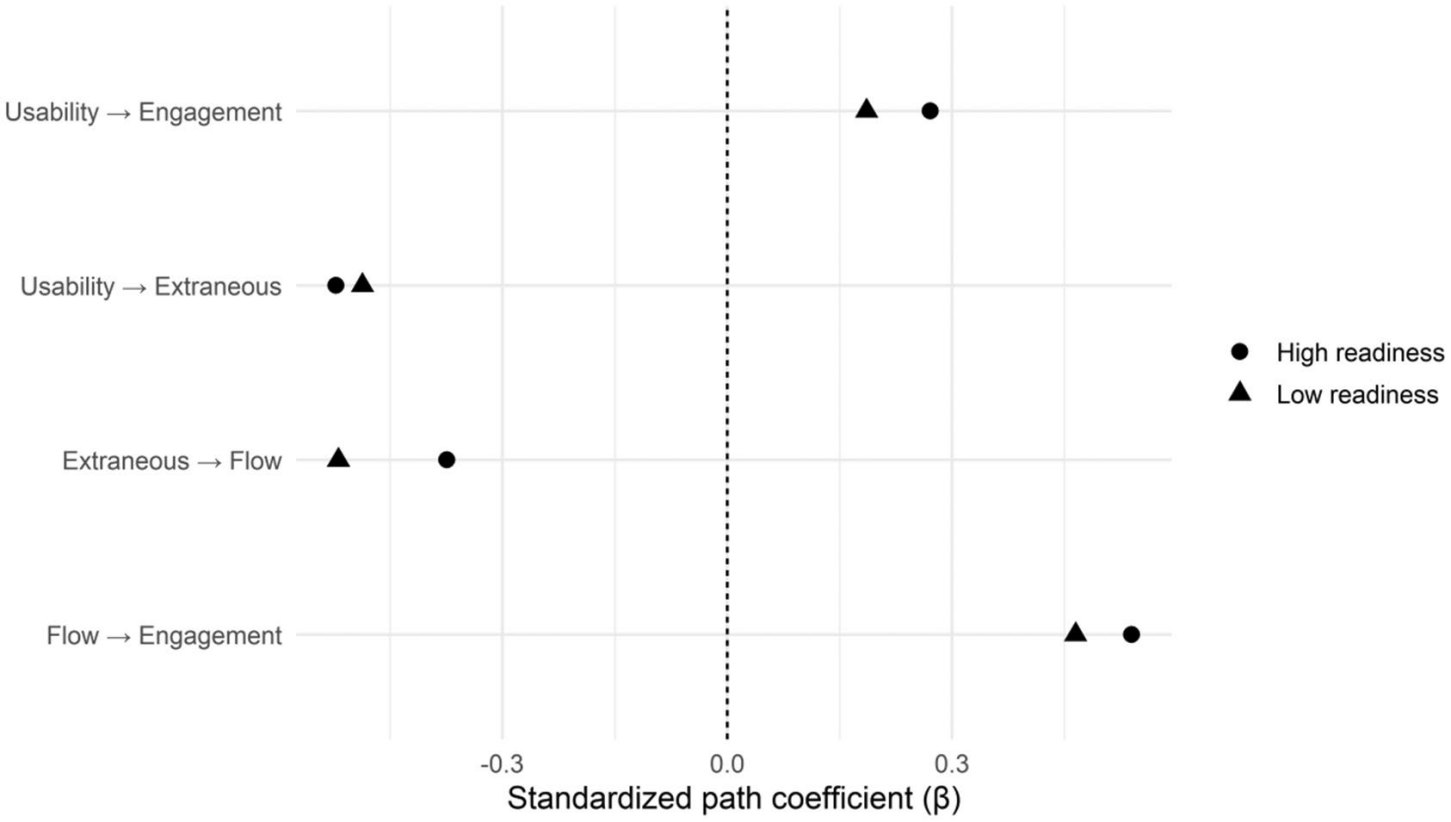
Comparison of standardized cross‐sectional association coefficients between high and low digital readiness groups.

The group comparison (Tables 7A and 7B and Figure 4) showed that the negative association between extraneous cognitive load and flow was stronger among students with lower digital readiness (*β* = −0.519) than among those with higher readiness (*β* = −0.374). This finding describes heterogeneity in a cross‐sectional association.

**TABLE 7A:** Results of measurement invariance tests.

Model	*χ* ^2^	df	CFI	TLI	RMSEA	SRMR	ΔCFI
Configural invariance	1025.46	1024	1.000	1.000	0.001	0.021	—
Metric invariance (loadings equal)	1046.551	1051	1.000	1.000	0.000	0.022	0.000
Scalar invariance (loadings + intercepts equal)	1063.93	1078	1.000	1.001	0.000	0.023	0.000

Abbreviations: CFI, comparative fit index; df, degree of freedom; RMSEA, root mean square error of approximation; SRMR, standardized root mean square residual; TLI, Tucker–Lewis index.

**TABLE 7B:** Comparison of group differences in core paths.

Hypothesis	Path	*β*_Low	*β*_High	CRD	*P*_CRD	*P*_*χ* ^*2*^ diff
H1	Usability → engagement	0.186	0.271	−1.295	0.195	0.188
H2	Usability → extraneous	−0.487	−0.522	0.907	0.364	0.309
H3	Extraneous → flow	−0.519	−0.374	−2.446	0.014	0.008
H4	Flow → engagement	0.465	0.540	−1.356	0.175	0.133

Abbreviation: CRD, critical ratio for differences.

To address the potential information loss associated with the median split, a supplementary moderation analysis treating digital readiness as a continuous variable was further conducted. The interaction term between extraneous cognitive load and standardized digital readiness was significantly associated with flow experience (*β* = 0.071, SE = 0.023, *p* = 0.002), indicating that the negative association was weaker at higher digital readiness between extraneous cognitive load and flow. Simple slope analysis showed that this negative association was stronger at low readiness (−1 SD: *b* = −0.52, *p* < 0.001) than at high readiness (+1 SD: *b* = −0.38, *p* < 0.001). These results were consistent with the multi‐group findings and were directionally consistent with the multi‐group findings.

## 4 Discussion

This section discusses the study findings in alignment with the simplified descriptive and inferential results presented above.

### 4.1 System Usability and Its Descriptive Foundation

The reverse‐scored mean usability item score was 3.256, equivalent to 56.4 on the conventional 0–100 SUS scale. In the structural model, usability was positively associated with learning engagement. Because the data were cross‐sectional, usability should be interpreted as a correlate and not as evidence of a temporal or causal relationship. In practice‐oriented nursing education, poorly designed systems may divert cognitive resources toward managing technical interactions rather than engaging with learning tasks, thereby undermining learning depth (Hyzy et al. 2022).

This observation is consistent with core assumptions of the TAM and related extensions (Dahleez et al. 2021), which emphasize the role of perceived ease of use in shaping engagement‐related behaviours. In simulation‐based nursing education, usability may therefore be considered alongside pedagogical quality when designing for learner engagement, particularly in environments characterized by high task complexity and repeated skill practice (Barz et al. 2024; Surbakti et al. 2024).

### 4.2 Sequential Cross‐Sectional Indirect Associations

The inferential analysis was consistent with a hypothesized sequence in which greater usability was associated with lower extraneous load, lower load was associated with greater flow, and greater flow was associated with engagement. This cross‐sectional indirect association does not demonstrate that usability first changed load or that load subsequently changed flow. Extraneous cognitive load was not directly associated with engagement after the other paths were included, suggesting that the observed covariance was represented mainly through flow in the specified model. Alternative temporal orderings remain possible (Skulmowski and Xu 2021; Merriweather et al. 2026; Faudzi et al. 2024). Instructional systems that impose unnecessary extraneous load occupy limited cognitive resources, constraining learners' ability to maintain focus on task‐relevant processes (Qi 2025). Flow theory further posits that optimal learning experiences emerge when learners can concentrate fully under conditions of clear goals, manageable challenge, and minimal distraction (Baxter et al. 2025). Notably, extraneous cognitive load did not directly predict learning engagement, suggesting that its influence is realized indirectly through its impact on learners' experiential states (Gutiérrez Maquilón et al. 2024; Sweller et al. 2019).

### 4.3 The Moderating Role of Digital Readiness

Our multi‐group analysis revealed that the negative association between extraneous cognitive load and flow was stronger among students with low digital readiness. Digital readiness varies substantially among nursing students, and AI systems designed primarily for digitally proficient users may be more difficult to use for students with lower readiness levels (Liu and Lin 2025). From an instructional design perspective, simplifying interface hierarchies, minimizing non‐essential operational steps, and providing graduated guidance may help limit avoidable cognitive demands and support equitable access to immersive learning experiences across diverse learner groups (Terry et al. 2019; Chen et al. 2025).

### 4.4 Theoretical Contributions and Limitations

By integrating cognitive load and flow theories, this study places the observed associations within a coherent theoretical framework. However, this study has several substantial methodological limitations. First, the cross‐sectional design precludes definitive causal inference; the observed associations are consistent with the hypothesized statistical indirect‐association model but cannot establish causality. Future longitudinal or experimental studies are strictly required. Although procedural remedies and statistical tests (Harman's single‐factor and ULMC) suggested CMB was not a severe threat, self‐reported data inherently carry common method variance risks. Moreover, the study lacks objective learning outcome measures. Subsequent research should incorporate objective performance indicators (e.g., OSCE scores and examination grades), subject to appropriate approvals and data‐protection procedures. Additionally, while the median‐split approach for moderation provided interpretable group comparisons, it may result in statistical information loss; however, the supplementary continuous‐variable interaction analysis reported in the Results yielded consistent conclusions, which mitigates this concern. Participants were reached through purposively selected questionnaire‐link dissemination channels at 16 colleges, but the colleges were not participating research institutions and no college or school identifiers were collected in the anonymous dataset. Accordingly, the findings should not be considered nationally representative. Digital readiness was a study‐specific exploratory index, and the nine‐item EduFlow‐2 adaptation requires fuller documentation.

### 4.5 Implications for Nursing Practice

Based on the findings of this study, several recommendations are proposed for nursing education practice and future research. First, nursing education institutions should prioritize the selection and adoption of AI virtual simulation systems with high usability, because usability was associated with learning engagement. Usability testing and iterative design improvements should be integrated into the procurement and implementation process of educational technology platforms. Second, instructional designers should consider clear navigation, concise instructions, and the avoidance of unnecessary operational steps because these features may limit extraneous cognitive demands and support flow during simulation practice. Third, differentiated support strategies should be developed for students with varying levels of digital readiness. Specifically, students with lower digital readiness may benefit from pre‐training orientation sessions, step‐by‐step guided tutorials, and additional technical support during initial system use in view of the stronger observed load–flow association in this group. Fourth, future research should employ longitudinal or experimental designs to establish causal relationships among the variables examined in this study. Additionally, incorporating objective learning outcome measures, such as Objective Structured Clinical Examination (OSCE) scores, system log data on usage patterns, and clinical competency assessments, would strengthen the evidence base. Finally, cross‐cultural validation studies are recommended to examine whether the observed sequential indirect‐association pattern generalizes across different educational and cultural contexts.

## 5 Conclusion

This study identified a hypothesized pattern of cross‐sectional associations in nursing simulation education: greater AI system usability was associated with lower extraneous cognitive load, greater flow experience, and higher learning engagement. A sequential statistical indirect association was observed, and the load–flow association differed by digital readiness. These results do not establish temporal ordering or causation; longitudinal or experimental research is needed.

## Author Contributions

**Mengjiao Liu:** conceptualization, data curation, formal analysis, investigation, methodology, project administration, writing – original draft. **Ping Zhang:** conceptualization, funding acquisition, methodology, project administration, resources, supervision, writing – review and editing. **Yeqing Wu:** data curation, formal analysis, investigation, visualization, writing – original draft. **Lu Pan:** data curation, formal analysis, validation, writing – review and editing. **Lan Li:** formal analysis, visualization, writing – original draft, data curation.

## Funding

This work was supported by the Natural Science Foundation of Hunan Province (Grand Number 2025JJ60785) and Department of Science and Technology of Hunan Province (Grand Number 2024JJ9226).

## Disclosure

The dataset comprising 2016 participants has not been reported in any previous publication and is not included in any other manuscript currently under consideration for publication.

## Ethics Statement

This study follows the ethical principles of the Helsinki Declaration and has been approved by the Ethics Committee of Xiangya Second Hospital, Central South University (approval number: LYEC2025‐0101). All participants must read and sign an informed consent form online before filling out the questionnaire, clarifying their understanding of the research purpose, voluntary participation nature, and the right to withdraw at any time. The survey collects data anonymously and does not involve any personally identifiable information. The data are only used for this study and is strictly stored in confidentiality to ensure that the privacy of participants is fully protected. Considering that this study is an anonymous questionnaire survey with extremely low risk, the ethics committee approves exemption from further ethical review requirements.

## Consent

The authors consents to the publication of this manuscript.

## Conflicts of Interest

The authors declare no conflicts of interest.

## Data Availability

The datasets generated during this study are available from the corresponding author upon reasonable request. Due to privacy protection requirements and ethical restrictions approved by the Ethics Committee of the Second Xiangya Hospital of Central South University (Approval No.: LYEC2025‐0101), raw data are not publicly available. Access to data requires a formal request and execution of a data use agreement to ensure compliance with the Declaration of Helsinki.
